# Interaction between drug and placebo effects: a cross-over balanced placebo design trial

**DOI:** 10.1186/1745-6215-11-110

**Published:** 2010-11-19

**Authors:** Muhammad M Hammami, Eman A Al-Gaai, Syed Alvi, Muhammad B Hammami

**Affiliations:** 1 Center for Clinical Studies and Empirical Ethics, King Faisal Specialist Hospital and Research Center, Riyadh, Saudi Arabia

## Abstract

**Background:**

The total effect of a medication is the sum of its drug effect, placebo effect (meaning response), and their possible interaction. Current interpretation of clinical trials' results assumes no interaction. Demonstrating such an interaction has been difficult due to lack of an appropriate study design.

**Methods:**

180 adults were randomized to caffeine (300 mg) or placebo groups. Each group received the assigned intervention described by the investigators as caffeine or placebo, in a randomized crossover design. 4-hour-area-under-the-curve of energy, sleepiness, nausea (on 100 mm visual analog scales), and systolic blood pressure levels as well as caffeine pharmacokinetics (in 22 volunteers nested in the caffeine group) were determined. Caffeine drug, placebo, placebo-plus-interaction, and total effects were estimated by comparing outcomes after, receiving caffeine described as placebo to receiving placebo described as placebo, receiving placebo described as caffeine or placebo, receiving caffeine described as caffeine or placebo, and receiving caffeine described as caffeine to receiving placebo described as placebo, respectively.

**Results:**

The placebo effect on area-under-the-curve of energy (mean difference) and sleepiness (geometric mean ratio) was larger than placebo-plus-interaction effect (16.6 [95% CI, 4.1 to 29.0] vs. 8.4 [-4.2 to 21.0] mm*hr and 0.58 [0.39 to 0.86] vs. 0.69 [0.49 to 0.97], respectively), similar in size to drug effect (20.8 [3.8 to 37.8] mm*hr and 0.49 [0.30 to 0.91], respectively), and its combination with the later was larger than total caffeine effect (29.5 [11.9 to 47.1] mm*hr and 0.37 [0.22 to 0.64]). Placebo-plus-interaction effect increased caffeine terminal half-life by 0.40 [0.12 to 0.68] hr (P = 0.007).

**Conclusions:**

Drug and placebo effects of a medication may be less than additive, which influences the interpretation of clinical trials. The placebo effect may increase active drug terminal half-life, a novel mechanism of placebo action.

**Trial Registration:**

ClinicalTrials.gov identification number - NCT00426010.

## Background

The placebo effect has been utilized in medical practice since antiquity and continues to be commonly used [1]. Recent systematic reviews [2,3] doubted the existence and clinical importance of a placebo effect despite evidence to the contrary [4-7], and more studies have been advocated to investigate its underlying mechanism(s) and clinical applications [8].

Comparing changes over time in the placebo arm of a clinical trial doesn't separate the placebo effect (meaning response) [4] from methodological factors such as regression to the mean, natural course, and the Hawthorne effect, and thus exaggerates the placebo effect [9], whereas comparing changes observed in the placebo arm to a no-treatment-control arm may underestimate the placebo effect since enrolled subjects know that they have only a 50% chance of getting a putatively active drug [5,10,11]. The balanced placebo design, where subjects receiving a test substance or a placebo are either informed that they are receiving this substance or placebo, or vice versa, can validly investigate the placebo effect [12] and has been used to study the placebo effect of several substances [13-18] including caffeine [13]. A modified balanced placebo model that uses a cross-over rather than parallel design has the advantage of reducing noise variation and sample size (which is ethically important in a design that employs deception) and permits the conduct of a standard bioequivalence study.

Few studies compared sizes of the placebo and drug components of a given medication effect or addressed their potential interaction [13,19-21]. Current interpretation of clinical trials assumes that there is no such interaction [21,22], and that the difference between medication and placebo arms represents drug effect rather than a combination of drug and interaction effects. Evaluating a potential interaction between drug and placebo effects allows testing the possibility that a placebo group may not be a good control group in clinical trials. Several studies have described various pharmacodynamic effects of placebos [13-18,23], however, the possibility that the placebo effect may involve modulation of drug bioavailability has not been explored. For example, it is theoretically possible that the placebo effect may involve altering gastric emptying, intestinal transit time, or drug elimination. Evaluating such possibility may provide insight into the mechanism of placebo and interaction effects as well as an objective outcome measure of the placebo effect.

Using a novel cross-over balanced placebo design, we measured the placebo effect on continuous and binary scales to: 1) explore potential interaction of drug and placebo effects by comparing the difference between receiving placebo described as caffeine or as placebo (placebo effect) to the difference between receiving caffeine described as caffeine or as placebo (placebo+interaction effect) and by comparing measured total medication effect to the sum of drug and placebo effects, 2) estimate the relative size of the placebo effect, and 3) explore the placebo effect on caffeine pharmacokinetics. Healthy subjects and caffeine were selected because of expected familiarity of caffeine effects to volunteers and of its safety profile as well as for ethical reasons, since the study design includes deception. We were able to measure an important placebo effect both on continuous and binary scales. Further, we found that the drug and placebo effects are less than additive and that the placebo effect may be due in part to modulation of the bioavailability of the active drug.

## Methods

### Study design

Participants were block-randomized (block size of four) to one of two randomized cross-over studies, one using caffeine described as caffeine or placebo and one using placebo described as placebo or caffeine. A 14 hour pharmacokinetics study was nested in the caffeine cross-over study. The wash-out time between the two periods of each balanced randomized cross-over study was 48 hours to allow for clearance of plasma caffeine [24] and most of caffeine (and caffeine withdrawal) effects [25], and minimize the occurrence of intra-subject differences. Balanced design was used to eliminate any differential residual carryover effect. There was no changes to methods after study commencement.

### Participants

Eligibility criteria for participants included an age of 18 to 50 years; being healthy, non-smoker, medication-free for one week, and able to reproducibly express oneself using a 100 mm visual analog scale (VAS); and daily caffeine consumption between 100 to 300 mg. The first was assessed by requesting participants to complete 7 VASs (on restlessness, irritability, flushing, headache, energy, sleepiness, and nausea) before and after obtaining their screening vital signs and without their knowledge of going to be retested. A difference of more than 10 mm on more than one VAS resulted in exclusion. The range of daily caffeine consumption was selected to minimize caffeine withdrawal symptoms and adverse effects (in caffeine-naïve subjects) and was estimated using the "Caffeine Content of Foods and Drugs" http://www.cspinet.org/new/cafchart.htm.

The study was conducted at the King Faisal Specialist Hospital and Research Center (KFSH & RC), Riyadh in February 2007 through February 2009, from 8-9 am to 12-1 pm. It followed published ethical guidelines on deception use in clinical research [7,8,22] and was approved by the Research Ethics Committee of KFSH & RC. All participants gave written consent being informed that the study is designed to compare the effects of capsules containing placebo or 300 mg caffeine (equivalent to 3 cups of brewed coffee), that the study aims to determine how much of the observed changes is not related to caffeine but to the placebo effect, and that they have a 50/50 chance of receiving a placebo or caffeine. They were given the choice to participate in the main 4-hour study or an extended 14-hour sub-study, and were compensated based on the Wage-Payment model [26]. At the completion of the entire study, and after obtaining their monetary compensation, they were contacted for briefing and a delayed consent, which was obtained from all the 95 contactable participants. None indicated that they have guessed the actual study aims. No adverse events (other than nausea) were noted.

### Interventions

Participants were requested to abstain from smoking and drinking alcohol for 2 days and from caffeine-containing beverages or food for 16 hours, before and throughout each study period, to fast from 9 pm, and to sleep for at least 8 hours.

To enhance the placebo effect, caffeine effects (and placebo-no-effect) were emphasized just before intervention and participants were requested to sign a statement to that effect (available in the supplement). Participants assigned to caffeine received 300 mg caffeine twice, 48 hours apart, one time dispensed from a bottle labelled "caffeine" and one time from a bottle labelled "placebo" in a random fashion. Participants assigned to placebo received placebo twice, 48 hours apart, one time dispensed from a bottle labelled "caffeine" and one time from a bottle labelled "placebo" in a random fashion. Caffeine and placebo were administered in the form of 2 capsules with 250 ml of water at ambient temperature, 15 to 30 minutes after a standardized light breakfast. Participants then abstained from food for four hours and remained ambulatory or seated upright until the end of the study. To enhance blinding and verify compliance, blood was drawn via intravenous cannula from all participants for caffeine level before and 3 hours after capsules' administration. For the 14-hour sub-study, additional blood samples were obtained at 0.25, 0.5, 0.75, 1, 1.25, 1.5, 1.75, 2, 2.5, 4, 6, 8, 10, 12, and 14 hours, and participants received standardized lunch and dinner that were the same in the two periods.

### Outcomes

Measurements were obtained before, and 0.5, 1.0, 1.5, 2.0, 2.5, 3.0, and 4.0 hours after intervention, in the following order: systolic blood pressure; VAS for energy, sleepiness, and nausea; and binary data for not-energetic, sleepy, and nauseated. Levels of energy, sleepiness, and nausea were self-measured on a 100 mm VAS anchored by word descriptors at each end (not at all, very). Caffeine levels were blindly measured by a locally-validated, modified high performance liquid chromatography assay [27]. The assay has a linear range of 0.05 to 20 μg/ml and an inter-run coefficient of variation and bias of ≤6.0% and ≤7%, respectively. Caffeine was stable in plasma (≥98%) for at least 12 weeks at -20°C. Plasma samples of each participant were stored in polypropylene tubes at -20°C, and analyzed together within 6 weeks.

Primary outcomes were 4-hour-area-under-the-curve (AUC_4_) of systolic blood pressure and VAS scores on self-measured energy, sleepiness, and nausea level in an analysis that was adjusted for baseline (analysis of covariance, ANCOVA). Secondary outcomes were binary measurements of subjective outcomes and placebo effect on caffeine pharmacokinetics. There were no changes to study outcomes after study commencement.

### Sample size

The study planned to recruit 90 subjects in each cross-over study, based on an expected standardized mean difference of placebo effect on continuous subjective outcome of 0.36[2], type I error of 0.05, type II error of 0.1, paired design, and 10% dropout rate.

### Randomization

The randomization schedule was generated by one of the authors (MMH) using a program available on-line http://www.randomization.com. Group assignment was concealed before randomization from participants and study coordinators who enrolled them. Interventions were assigned to participants by two of the investigators (MMH and EAG).

### Blinding

Study coordinators who collected data were blinded to study design and participants' assignments. Caffeine levels were determined by one of the authors (SA) who was blinded to study design and participants' assignment. Participants were deceived as to the true aim of the study and to their assignment.

### Statistical methods

Because of positive skew, the AUC_4 _values for sleepiness and nausea levels were analyzed after natural logarithmic transformation then back transformed, so that the difference between the groups is expressed as geometric means ratio. The ANCOVA model included group and subjects nested within groups (as appropriate), period, intervention, and baseline value. Mean percentage of time participants reported being not-energetic, sleepy, or nauseated was evaluated by the t test. Analyses were conducted by one author (MMH) with SPSS for Windows software (release 16.0.0, 2007. SPSS Inc., Chicago, ILL, USA) and SAS for Windows software (version 9.2, SAS Institute Inc., Cary, NC, USA). 2-tailed p values are reported unless indicated otherwise.

### Model

**Figure 1:**
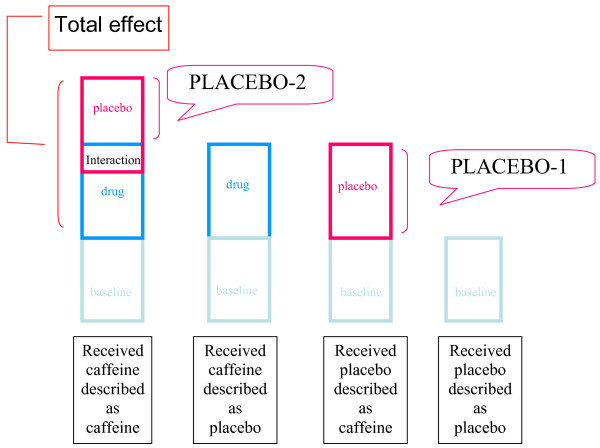
**Model of drug and placebo effects and their interaction**. Total effect is inferred from the difference between received caffeine described as caffeine and received placebo described as placebo. Drug effect is inferred from the difference between received caffeine described as placebo and received placebo described as placebo. The model predicts two kinds of measured "placebo effect": a) PLACEBO-1 is inferred from the difference between received placebo described as caffeine and received placebo described as placebo, which contains the placebo effect only, and b) PLACEBO-2 is inferred from the difference between received caffeine described as caffeine and received caffeine described as placebo, which contains the placebo effect and the interaction effect. Note that PLACEBO-1 is depicted here larger than PLACEBO-2 and that the total effect is smaller than the combination of the drug and placebo effects (as measured by PLACEBO-1) to indicate that the drug and placebo effects are less than additive.

We assumed that outcome measures associated with receiving placebo described as placebo represent baseline value, including non-specific changes, outcome measures associated with receiving placebo described as caffeine represent placebo effect + baseline value, outcome measures associated with receiving caffeine described as placebo represent drug effect + baseline value, and outcome measures associated with receiving caffeine described as caffeine represent drug effect + placebo effect + interaction effect (drug effect * placebo effect) + baseline value. Drug effect was estimated by comparing receiving caffeine described as placebo to receiving placebo, described as placebo, placebo effect by comparing receiving placebo described as caffeine to receiving placebo described as placebo, placebo+interaction effect by comparing receiving caffeine described as caffeine to receiving caffeine described as placebo, and total effect by comparing receiving caffeine described as caffeine to receiving placebo described as placebo. Total effect would include drug and placebo effects and their interaction. The model is presented in Figure 1.

## Results

**Figure 2:**
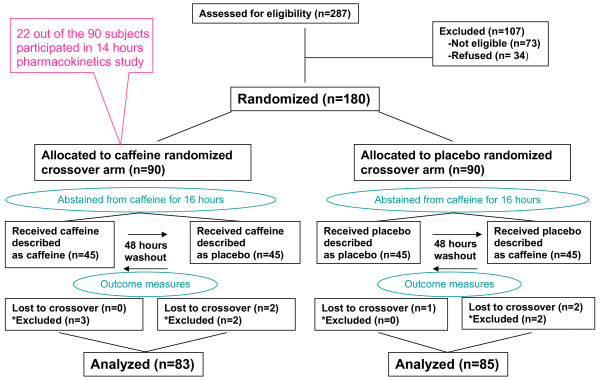
**Flow diagram of study procedures**. *Excluded because of failure to abstain from caffeine as reflected on baseline level.

We screened 287 volunteers and found 214 eligible. Ineligibility was related to medical history (17), blood test results (12), daily caffeine intake (11), heavy smoking (6), and education level/VAS test (27). 34 of the 214 eligible volunteers did not show up for the study, thus 180 were equally randomized to caffeine or placebo cross-over arms. We excluded from analysis participants who later withdrew from the study (3 randomized to placebo, 2 to caffeine) or did not adequately abstain from caffeine (baseline caffeine levels in the study periods differed by ≥1 μg/ml (2 randomized to placebo and 5 to caffeine). A flow chart is presented in Figure 2. Study coordinators guessed that 52%, 51%, 41%, and 44% of participants who received, caffeine described as caffeine, caffeine described as placebo, placebo described as placebo, and placebo described as caffeine, respectively, received caffeine; indicating the success of blinding. Baseline characteristics of study groups are shown in Table 1.

**Table 1:** Baseline characteristics of study participants.

Characteristics	Caffeine Group* (N = 83)		Placebo Group† (N = 85)		Both Groups (N = 168)
Age - mean (SD), yr	30.6 (6.6)		28.3 (5.7)		29.4 (6.4)
Sex - no. (%)					
Female	10 (12)		17 (20)		27 (16)
Male	73 (88)		68 (80)		141 (84)
Completed education - no. (%)					
High school	15 (18)		7 (8)		22 (13)
College	44 (53)		54 (64)		98 (58)
University	24 (29)		24 (28)		48 (29)
Caffeine consumption - no. (%)					
100-149 mg/dy	28 (34)		23 (27)		51 (30)
150-199 mg/dy	28 (34)		24 (28)		52 (31)
200-249 mg/dy	15 (18)		22 (26)		37 (22)
250-300 mg/dy	12 (14)		16 (19)		28 (17)
Occupational Status - no. (%)					
Professional, technical, managerial	17 (21)		19 (22)		36 (22)
Clerical, sales	24 (29)		32 (38)		56 (34)
Service	22 (27)		25 (29)		47 (28)
Agricultural, fishery related	10 (12)		1 (1)		11 (7)
Students	6 (7)		8 (9)		14 (8)
Unemployed	3 (3)		0 (0)		3 (2)
Race or ethnic group - no. (%)					
Saudi	3 (4)		9 (11)		12 (7)
Arab, non-Saudi	14 (17)		11 (13)		25 (15)
Asian	66 (80)		65 (76)		131(78)
	**Overt**	**Covert**	**Overt**	**Covert**	
Caffeine level - mean (SD), μg/ml					
Baseline	0.26 (0.43)	0.20 (0.34)	0.30 (0.56)	0.29 (0.35)	
3-hour	9.38 (2.86)	9.08 (2.77)	0.17 (0.25)	0.18 (0.24)	
Energy level - mean (SD), mm‡	71 (25)	70 (24)	74 (19)	74 (20)	
Sleepiness level - mean (SD), mm‡	19 (22)	22 (26)	20 (25)	20 (20)	
Nausea level - mean (SD), mm‡	7 (14)	6 (13)	8 (18)	9 (15)	
Systolic blood pressure - mean (SD), mm Hg	121 (14)	122 (13)	121 (12)	122 (12)	

*Received 300 mg caffeine twice, described as caffeine (overt) or as placebo (covert) in a balanced randomized cross-over design. †Received placebo twice, described as placebo (overt) or as caffeine (covert) in a balanced randomized cross-over design. ‡ Measured on 100 mm visual analogue scale.

### Placebo effect on subjective endpoints

**Figure 3:**
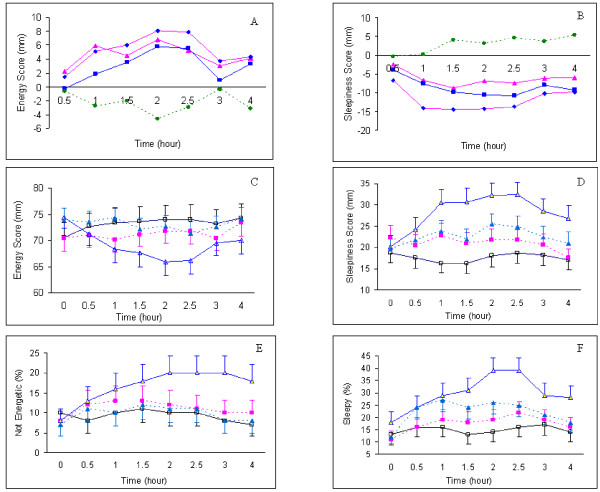
**Self-reported energy and sleepiness levels on continuous and binary scales over four hours after intervention**. A and B: Time course of estimated unadjusted total effect (closed diamond), drug effect (closed squares), placebo effect (closed triangles), and interaction effect (closed circles with interrupted line) on VAS scores of energy and sleepiness level, respectively. C and D: Mean unadjusted VAS Scores for energy and sleepiness, respectively. E and F: Mean unadjusted percentage of time, lack of energy and sleepiness, respectively, were reported. T bars indicate standard errors. Squares indicate receiving 300 mg caffeine, described as caffeine (open square with continuous line) or as placebo (closed square with interrupted line) by 83 subjects in a balanced randomized cross-over design. Triangles indicate receiving placebo described as placebo (open triangle with continuous line) or as caffeine (closed triangle with interrupted line) by 85 subjects in a balanced randomized cross-over design. The difference between open squares and open triangles represents the total effect. The difference between closed squares and open triangles represents the drug effect. The difference between open triangles and closed triangles represents the placebo effect. The difference between open squares and closed squares represents the placebo+interaction effect.

The estimated drug, placebo, and total effects are presented in Figure (3A &3B) and Table 2. There was significant placebo effect on energy level, and placebo effect and placebo+interaction effect on sleepiness level. The placebo effect on energy and sleepiness levels and placebo+interaction effect on sleepiness level were still significant using Wilcoxon Signed Ranks test without adjustment for baseline (P = 0.03, P = 0.01, and P = 0.03, respectively), indicating robustness of the results. There was significant correlation between placebo or placebo+interaction effects on energy and sleepiness (placebo effect: r = -0.62, p < 0.001; rho = -0.64, p < 0.001 and placebo+interaction effect: r = -0.62, p < 0.001; rho = -0.58, p < 0.001). There was no significant placebo or placebo+interaction effect on nausea.

**Table 2:** Drug, placebo, placebo+interaction, and total effects on systolic blood pressure, energy, sleepiness, and nausea levels.

	Drug Effect	Placebo Effect	Placebo+ interaction Effect	Total Effect
**Energy level** (mm*hr)	20.8 [3.8 to 37.8] P = 0.02	16.6 [4.1 to 29.0] P = 0.01	8.4 [-4.2 to 21.0] P = 0.19	29.5 [11.9 to 47.1] P = 0.001
**Sleepiness level** (ratio)	0.49 [0.30 to 0.91] P = 0.005	0.58 [0.39 to 0.86] P = 0.007	0.69 [0.49 to 0.97] P = 0.03	0.37 [0.22 to 0.64] P < 0.0001
**Nausea level** (ratio)	1.59 [1.00 to 2.52] P = 0.05	1.43 [0.91 to 2.27] P = 0.12	1.04 [0.74 to 1.47] P = 0.82	1.46 [0.91 to 2.34] P = 0.12
**Systolic blood pressure** (mm Hg*hr)	28.4 [20.5 to 36.4] P < 0.0001	1.2 [-3.0 to 5.4] P = 0.57	-0.2 [-4.6 to 4.1] P = 0.91	29.9 [22.3 to 37.6] P < 0.0001

Data are adjusted point estimate [95% confidence interval]. Point estimate is the difference between means (systolic blood pressure and energy levels) or ratio of geometric means (sleepiness and nausea levels). Drug effect = "receiving caffeine described as placebo" mean - "receiving placebo described as placebo" mean or ratio of their geometric means. Placebo effect = "receiving placebo described as caffeine" mean - "receiving placebo described as placebo" mean or ratio of their geometric means. Placebo+interaction effect = "receiving caffeine described as caffeine" mean - "receiving caffeine described as placebo" mean or ratio of their geometric means. Total effect = "receiving caffeine described as caffeine" mean - "receiving placebo described as placebo" mean or ratio of their geometric means. Unadjusted mean(SE) of energy (mm*hr), sleepiness (mm*hr), nausea (mm*hr), and systolic blood pressure (mm Hg*hr) levels were respectively, 293.5(10.1), 70.3(8.5), 23.8(4.3), and 499(5.2) when caffeine was described as caffeine; 284.9(8.9), 83.5(8.7), 23.8(4.7), and 500(5.1) when caffeine was described as placebo; 275.2(9.2),114.8(10.5), 23.7(5.1), and 469(4.3) when placebo was described as placebo; and 291.8(6.6), 91.1(8.3), 24.1(3.7), and 471(4.7) when placebo was described as caffeine.

Figure (3C &3D) depicts unadjusted mean (SE) VAS scores of energy and sleepiness determined over 4 hours after intervention. Participants also provided binary answers about feeling not-energetic, sleepy, or nauseated, at zero time and at 0.5, 1, 1.5, 2, 2.5, 3, and 4 hours after intervention (Figure (3E &3F)). The binary data were consistent with the continuous data. Using percentage of time symptoms were reported over 4 hours as a summary measure, there was significant placebo effect on feeling not-energetic and sleepy (Table 3) that was still significant using Wilcoxon Signed Ranks test (1-tailed P = 0.006 and P = 0.04, respectively). The placebo+interaction effect was not significant.

**Table 3:** Drug, placebo, placebo+interaction, and total effects on self-classification as not-energetic, sleepy, and nauseated.

	Drug Effect	Placebo Effect	Placebo+ interaction Effect	Total Effect
Not-energetic	-6.3 [-15.4 to 2.8] P = 0.09	-7.9 [-14.5 to -1.3] P = 0.01	-2.4 [-7.8 to 3.0] P = 0.19	-8.6 [-17.5 to -1.7] P = 0.03
Sleepy	-12.9 [-23.1 to -2.6] P = 0.007	-7.9 [-16.4 to 0.0] P = 0.03	-3.1 [-10.4 to 4.1] P = 0.19	-16.0 [-26.2 to -5.8] P = 0.001
Nauseated	-2.1 [-8.1 to 3.8] P = 0.24	-3.4 [-8.8 to 1.9] P = 0.10	1.2 [-3.8 to 6.3] P = 0.32	-0.9 [-7.3 to 5.5] P = 0.39

Data are mean [95% confidence interval] percentage of time the particular symptom was reported over 4 hours after intervention.

P values are from 1-tailed t test. Drug effect = "receiving caffeine described as placebo" mean - "receiving placebo described as placebo" mean. Placebo effect = "receiving placebo described as caffeine" mean - "receiving placebo described as placebo" mean. Placebo+interaction effect = "receiving caffeine described as caffeine" mean - "receiving caffeine described as placebo" mean. Total effect = "receiving caffeine described as caffeine" mean - "receiving placebo described as placebo" mean. Mean(SE) percentage of time not-energetic, sleepy, or nauseated, respectively, were reported over 4 hours after intervention were 10.0(2.6), 15.9(3.2), and 7.6(2.1) when caffeine was described as caffeine; 12.4(2.9), 19.0(3.3), and 6.3(1.7) when caffeine was described as placebo; 18.6(3.6), 31.9(4.1), and 8.5(2.4) when placebo was described as placebo; and 10.8(2.7), 24.0(3.5), and 5.0(1.1) when placebo was described as caffeine.

The placebo and drug effects were comparable in size on the three subjective endpoints (Tables 2 &3). Head to head comparison of the ANCOVA-adjusted means of the placebo and drug effects, using t test did not show a significant difference (p = 0.66 to 0.75).

### Interaction between drug and placebo effects

As shown in Table 2, the placebo effect was larger than the placebo+interaction effect on energy and sleepiness levels, and the combination of drug and placebo effects was larger than the total effect. Further, while the placebo effect was significant on binary endpoints, the placebo+interaction effect was not (Table 3). We also estimated the drug+interaction effect by comparing receiving caffeine described as caffeine to receiving placebo described as caffeine, using ANCOVA. The drug+interaction effect was not significant on energy level 11.3 [CI, -4.3 to 27.0] mm*hr, p = 0.15) and of borderline significance on sleepiness level (geometric mean ratio 0.56 [CI, 0.32 to 1.00), p = 0.05). The data combined strongly suggest the presence of a negative interaction between caffeine drug and placebo effects.

Drug and placebo effects followed similar time course with a peak at 2 to 2.5 hours, whereas, the interaction effect on sleepiness level appeared to increase over the study period (Figure (3A &3B)).

### Placebo effect on objective outcomes

**Figure 4:**
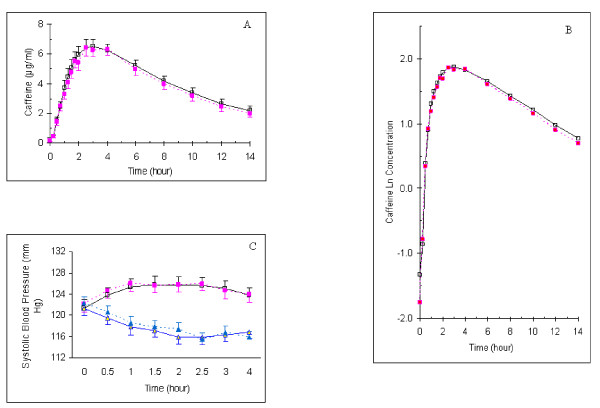
**Placebo effect on caffeine plasma levels and systolic blood pressure**. A & B: Mean plasma caffeine levels over 14 hours after administration of caffeine to 22 participants in a balanced cross-over design before (A) and after natural logarithmic transformation (B). C: Systolic blood pressure over four hours following intervention. Squares indicate receiving 300 mg caffeine described as caffeine (open square with continuous line) or as placebo (closed square with interrupted line). Triangles indicate the administration of placebo described as placebo (open triangle with continuous line) or as caffeine (closed triangle with interrupted line). T bars indicate standard errors.

We examined whether caffeine plasma level depends upon whether participants knew they were getting caffeine. We conducted a 14 hour bioavailability study on 22 participants nested in the 83 who received caffeine described as caffeine or as placebo in a balanced randomized cross-over design, under controlled food and fluid intake. Mean plasma caffeine levels before and after natural logarithmic transformation are shown in Figure (4A &4B). They were lower in the terminal part of the curve after receiving caffeine described as placebo compared to receiving caffeine described as caffeine. Caffeine pharmacokinetics is shown in Table 4. Using ANOVA (model included group, subjects nested in groups, period, and intervention), there was no significant difference in maximum measured plasma level or its time. However, caffeine AUC was significantly lower and caffeine terminal half-life was significantly shorter after receiving caffeine described as placebo. These differences continued to be significant using ANOVA after logarithmic transformation of data or Wilcoxon Signed Ranks test (Table 4). There was no significant period (p = 0.45 to 0.80) or group effect (p = 0.47 to 0.79). Furthermore, there was a significant difference in mean 3-hour caffeine level (Table 1) in the entire group of 83 participants who received caffeine when caffeine was described as caffeine compared to when it was described as placebo (0.3 [CI, 0.04 to 0.57] μg/ml, p = 0.02).

**Table 4:** Pharmacokinetics of caffeine when described as caffeine or placebo.

	Untransformed					Log-transformed			
	AUC_14 _(μg/ml*hr)	AUC_∞_ (μg/ml*hr)	C_max_ (μg/ml)	T_max_ (hr)	t _1/2_ (hr)	AUC_14_ (μg/ml*hr)	AUC_∞_ (μg/ml*hr)	C_max_ (μg/ml)	t _1/2_ (hr)
Described as Caffeine*	58.4 (4.6)	82.8 (9.6)	7.2 (0.5)	2.9 (0.3)	6.3 (0.5)	4.01 (0.08)	4.30 (0.10)	1.93 (0.07)	1.77 (0.08)
Described as Placebo*	56.2 (4.3)	77.4 (8.7)	7.1 (0.4)	2.7 (0.2)	5.92 (0.5)	3.97 (0.07)	4.24 (0.10)	1.93 (0.06)	1.71 (0.08)
ANOVA†	2.33 [0.35 to 4.31] P = 0.02	5.43 [1.62 to 9.23] P = 0.008	0.06 [-0.25 to 0.37] P = 0.70	0.22 [-0.33 to 0.77] P = 0.42	0.40 [0.12 to 0.68] p = 0.007	1.04 [1.00 to 1.07] P = 0.05	1.07 [1.02 to 1.11] P = 0.004	1.00 [0.94 to 1.06] P = 0.99	1.07 [1.02 to 1.12] P = 0.01
Wilcoxon Signed Ranks‡	P = 0.02	P = 0.006	P = 0.61	P = 0.46	P = 0.007				

*Data are unadjusted mean (SE) before and after natural logarithmic transformation. †Data are adjusted point estimate [95% confidence interval]. Point estimate is "receivng caffeine described as caffeine" mean - "receivng caffeine described as placebo" mean (untransformed data) or ratio of their geometric means (transformed data). The ANOVA model included group, subjects nested in groups, period, and intervention. There was no significant period or group effects (P = 0.45 to 0.80). ‡Comparing "receiving caffeine described as caffeine" to "receiving caffeine described as placebo". AUC_14_, area under the plasma concentration-time curve from time 0 to 14 hour (calculated by linear trapezoidal method); AUC_∞, _area under the plasma concentration-time curve from time 0 to infinity (AUC_4 _+ last measured level/terminal rate constant); C_max _and T_max_, maximum measured plasma level and its time; t_1/2_, plasma half-life (Ln 2/terminal elimination constant [slope of the linear regression line of natural log-transformed last 6 measurable caffeine levels vs time curve]).

We did not find a significant placebo effect or placebo+interaction effect on systolic blood pressure despite the presence of a clear drug effect (Table 2 and Figure 4(C)).

## Discussion

Our study design allowed dissecting total caffeine effect into its components: drug, placebo, and interaction effects. It was argued that if a placebo effect exists it would be of negligible importance [2]. We found that caffeine drug and placebo effects were similar in size, consistent with a recent analysis of 37 trials in which patients were randomized to no treatment, placebo, or active intervention, that found the relative contributions of spontaneous improvement and of placebo to that of active interventions, 24% and 20%, respectively [23]; and the observation that patients' belief [28] and preference [29] regarding treatment assignment were clinically important; and suggesting the worth of investing more time and effort on maximizing placebo benefits in clinical practice. The observed placebo effect size may explain why generic drug formulations that pass rigorous bioequivalence studies are seen as less potent (not more potent) than their more expensive branded counterparts [30,31].

The placebo effect was larger when measured using placebo (described as caffeine or as placebo) compared to when measured using caffeine (described as caffeine or as placebo), the combination of placebo and drug effects was larger than total effect, and the combination of drug and interaction effects was smaller than drug effect. Although these differences were not statistically significant, together they suggest that caffeine drug and placebo effects are less than additive. In previous studies that used a balanced placebo design, caffeine placebo [32] and alcohol placebo [20] effects were also more readily seen using placebo than using the active substance. Further, a neuro-imaging study showed that alcohol intoxication and expectancy have opposite effects on neuronal activation [14]. The possibility that there may be an interaction between placebo and drug effects has important implications. The difference between drug and placebo arms in clinical trials may represent not only drug pharmacological effect but also an interaction effect and thus may underestimate (if effects are less than additive) or overestimate (if effects are more than additive) pharmacological drug effect. The possibility of less than additive effects may explain the clinically trivial effect of antidepressants as deduced from clinical trials [21].

Converging evidence suggests that different mechanisms may underlie different placebo effects, for example, opioid pathways underlie placebo analgesia and dopaminergic pathways underlie placebo effect on movement disorders [7,33]. We tested a priori hypothesis that placebo effect may influence drug bioavailability. We found no effect on the rate of caffeine absorption but significant and unexpected effect on caffeine terminal half-life, and as a result, caffeine AUC (about 7% increase). In addition to providing evidence for placebo effect on objective endpoints, this observation presents a novel mechanism of placebo effect that deserves further studies, and suggests the importance of blinding in bioequivalence studies that compare generic to brand drug formulations and in clinical trials with objective endpoints.

Study limitations include intervention's administration by an undeceived investigator which may have reduced the placebo effect. Further, the balanced placebo design assumes that instructions fully control participants' beliefs regarding their assignment, which we did not verify; and is prone to experimental subordination bias [22], which can inflate the placebo response. However, the absence of a physician-patient relationship and of placebo effect on nausea, and the different sizes of the placebo effect in the two arms of the study suggest that the effect of such bias would be minimal.

## Conclusions

In this large randomized, cross-over balanced placebo design study, using caffeine as a model drug, we found that: 1) a significant placebo effect could be measured on continuous and binary scales, 2) the placebo effect is similar in size to the drug effect, 3) drug and placebo effects may be less than additive, and 4) the placebo effect may be due in part to modulation of drug metabolism.

## Competing interests

The authors declare that they have no competing interests.

## Authors' contributions

MMH conceived of the study, designed the study, performed pharmacokinetic and statistical analysis, conducted literature review, and wrote the manuscript. EAG participated in data acquisition and literature review. SA developed the caffeine assay and analyzed caffeine levels. MBH participated in statistical analysis, literature review, and writing of the manuscript. All authors read and approved the final manuscript.
